# Transactions of the Chicago Society of Physicians and Surgeons, October 26, 1874

**Published:** 1874-11-15

**Authors:** Ralph E. Starkweather


### Society Reports.

### TRANSACTIONS OF THE CHICAGO SOCIETY OF PHYSICIANS AND SURGEONS.

### REGULAR MEETING, OCTOBER 26, 1874.

*Reported by Ralph E. Starkweather, M.D.*

DR. BARTLETT, President, in the Chair. After the reading of the minutes of the previous meeting the name of Dr. R. L. Leonard was favorably reported to the Society by the Board of Censors and received an unanimous election.

The Society listened to the reading of a translation from the German of Doctors Kundrat, of Vienna, and Englemann, of St. Louis, on "The Microscopical Appearances of the Uterine Mucous Membrane," made and read by Dr. Von Mansfelde. At its conclusion a vote of thanks was carried, but, owing to the length of time occupied by the reading of the paper, upwards of two hours, its discussion was deferred.

The special committee on the Relations between Physicians and Pharmacists, through its chairman, Dr. Hyde, reported as follows:

*Mr. President and Gentlemen:*

The committee of your appointment, designed to co-operate with others from the Chicago Medical Society, and the Chicago College of Pharmacy, have the honor to submit herewith the unanimous report of the Joint Committee on the subject assigned to them for investigation.

The time which has elapsed since the date of our appointment has not been an indication of any negligence on the part of your representatives. It was found to be necessary to hold several meetings in order to arrive at definite conclusions, to which all might assent; and these meetings have served to elicit the opinions of every member of the Committee on the questions at issue, in the somewhat protracted discussions to which they have given rise.

This Society has already pronounced such an unqualified and unanimous condemnation of every course which involves the acceptance of an obligation by physicians from pharmacists, that further action in the premises would seem to be superfluous. We merely call your attention to those features in the subjoined recommendation which have not yet been brought to your notice.

Your Committee has been united in the propositions set forth in the Joint Committee. Either the practices referred to are reprehensible or commendable. Abundant proof has been laid before us to the effect that the practices deprecated have been pursued by reputable practitioners in this city. And it is to us a matter of very great surprise and regret that they should be viewed in one light by one medical society, and in another and very different light by the other, as the press notices of the action of our sister organization would make it appear. We would urge upon you by every consideration of professional honor, the establishment of a high and unalterable standard of ethics, which shall in its turn elevate those who are willing to conscientiously and fearlessly adhere to it.

[Signed,] JAMES H. ETHERIDGE,
JAMES NEVINS HYDE,
WALTER HAY.

The following is the Joint Report:

We, the undersigned, members of a Joint Committee, consisting of delegates from the Chicago Medical Society, the Chicago College of Pharmacy, and the Chicago Society of Physicians and Surgeons, agree to report to our respective organizations a recommendation of resolutions condemning the following practices:

*First.*—The payment of commissions by pharmacists to physicians, in the form of nominal or free office rental, money, or perquisites.

*Second.*—The practice of any branch of medicine by pharmacists.

*Third.*—The use of prescription blanks bearing the name of a pharmacist.

*Fourth.*—The prescribing of medicines by adding to their titles that of a proprietor or patentee.

*Fifth.*—The use of private formulæ by which certain pharmacists, exclusively, are enabled to compound prescriptions.

*Resolved*, That we fully endorse the project of the Chicago College of Pharmacy, by which it is proposed to prepare and publish a codex or collection of formulæ, according to which any physician may order, and any pharmacist prepare, desirable compounds not enumerated in the United States Dispensatory, with greater convenience and uniformity; such codex being subject at any time to addition and revision by the organization here named.

The report was accepted. After a long and exhaustive discussion, in which a large number participated, the report was adopted by ayes and nays, some of the members declining to vote. The attendance at the meeting was one of the largest of the present year. The motion to reconsider the vote adopting the report was laid upon the table till the next regular meeting. At a very late hour the Society adjourned.

